# OutcropHyBNet: Hybrid Backbone Networks with Data Augmentation for Accurate Stratum Semantic Segmentation of Monocular Outcrop Images in Carbon Capture and Storage Applications

**DOI:** 10.3390/s23218809

**Published:** 2023-10-29

**Authors:** Hirokazu Madokoro, Kodai Sato, Stephanie Nix, Shun Chiyonobu, Takeshi Nagayoshi, Kazuhito Sato

**Affiliations:** 1 Faculty of Software and Information Science, Iwate Prefectural University, Takizawa 020-0693, Japan; 2 Faculty of Systems Science and Technology, Akita Prefectural University, Yurihonjo 015-0055, Japan; 3 Graduate School of International Resource Sciences, Akita University, Akita 010-8502, Japan; 4 Faculty of Bioresource Sciences, Akita Prefectural University, Akita 010-0195, Japan

**Keywords:** semantic segmentation, Convolutional Neural Network, Vision Transformer, Generative Adversarial Networks, outcrop images, drone

## Abstract

The rapid advancement of climate change and global warming have widespread impacts on society, including ecosystems, water security, food production, health, and infrastructure. To achieve significant global emission reductions, approximately 74% is expected to come from cutting carbon dioxide (CO${}_{2}$) emissions in energy supply and demand. Carbon Capture and Storage (CCS) has attained global recognition as a preeminent approach for the mitigation of atmospheric carbon dioxide levels, primarily by means of capturing and storing CO${}_{2}$ emissions originating from fossil fuel systems. Currently, geological models for storage location determination in CCS rely on limited sampling data from borehole surveys, which poses accuracy challenges. To tackle this challenge, our research project focuses on analyzing exposed rock formations, known as outcrops, with the goal of identifying the most effective backbone networks for classifying various strata types in outcrop images. We leverage deep learning-based outcrop semantic segmentation techniques using hybrid backbone networks, named OutcropHyBNet, to achieve accurate and efficient lithological classification, while considering texture features and without compromising computational efficiency. We conducted accuracy comparisons using publicly available benchmark datasets, as well as an original dataset expanded through random sampling of 13 outcrop images obtained using a stationary camera, installed on the ground. Additionally, we evaluated the efficacy of data augmentation through image synthesis using Only Adversarial Supervision for Semantic Image Synthesis (OASIS). Evaluation experiments on two public benchmark datasets revealed insights into the classification characteristics of different classes. The results demonstrate the superiority of Convolutional Neural Networks (CNNs), specifically DeepLabv3, and Vision Transformers (ViTs), particularly SegFormer, under specific conditions. These findings contribute to advancing accurate lithological classification in geological studies using deep learning methodologies. In the evaluation experiments conducted on ground-level images obtained using a stationary camera and aerial images captured using a drone, we successfully demonstrated the superior performance of SegFormer across all categories.

## 1. Introduction

The escalating global phenomenon of climate change, resulting from the warming of the earth, has reached a level of utmost urgency. In its Second Working Group Report of the Sixth Assessment Report [1], the Intergovernmental Panel on Climate Change (IPCC) highlighted the profound impact of climate change on various human systems, including ecosystems, water security, food production, health and well-being, cities, residences, and infrastructure. According to the First Working Group Report, the global average temperature from 2011 to 2020 has risen by 1.09 °C compared to the pre-industrial era. Furthermore, the IPCC announced that even under scenarios with extremely low greenhouse gas emissions, such as achieving zero carbon dioxide (CO${}_{2}$) emissions by around 2050 or later and subsequent negative emissions, there is a possibility of global temperature increase reaching 1.5 °C between 2021 and 2040. The report also indicated that the frequency of extreme temperature events in terrestrial areas that occur once every 10 years or once every 50 years is likely to increase by 4.1 times and 8.6 times, respectively, due to 1.5 °C of warming. In addition to the projected increase of 1.5-fold in decadal events for heavy rainfall in terrestrial areas and a 2.0-fold increase in agricultural and ecological droughts in arid regions, it is anticipated that severe snowstorms and super typhoons will undergo further intensification [2].

The First Working Group Report revealed a nearly linear relationship between cumulative CO${}_{2}$ emissions and the phenomenon of global warming. To limit the temperature increase beyond the pre-industrial levels to 1.5 °C with a probability of 67% or higher, it was estimated that the remaining CO${}_{2}$ emissions should not exceed 400 billion tons. The Third Working Group Report stated that in scenarios where global CO${}_{2}$ emissions reach zero, approximately 74% of the required global emissions reduction would be achieved through reductions in CO${}_{2}$ emissions from energy supply and demand. While renewable energy has emerged as a prominent solution, it is recognized that a combination of renewable energy sources and fossil fuel systems is still necessary to meet the current energy demand. In light of this, the present study specifically focuses on carbon capture and storage (CCS) technology [3], assuming a high carbon capture rate of 90–95% from fossil fuel systems.

Achieving carbon neutrality requires a balance between emissions and removals of greenhouse gases [4]. However, in many sectors, complete decarbonization is proving to be a challenging reality. A prime example of this challenge is the power generation sector. In this context, CCS technology plays an indispensable role in effectively reducing CO${}_{2}$ emissions and achieving the goal of carbon neutrality. The automotive industry is also progressing towards decarbonization. The transition from internal combustion engines to electric motors has led to a reduction in CO${}_{2}$ emissions. However, charging the batteries of electric vehicles requires a substantial amount of electricity, and relying solely on renewable energy sources to meet this demand presents a formidable challenge. Nuclear power as an alternative energy source remains a subject of debate, and its utilization presents significant challenges. These challenges include the management of nuclear waste, the threat of terrorism, and the need to learn from past nuclear power plant accidents while undertaking long-term decommissioning processes, which are complex and require careful consideration. To achieve carbon neutrality, a diverse array of strategies and approaches is imperative.

CCS refers to the collective techniques of capturing carbon dioxide emitted from factories, power plants, and other sources and storing it underground before its release into the atmosphere [5]. The selection of storage locations is based on geological models derived from borehole surveys and probability statistics. However, the current statistical methods used to create geological models from limited sampling information obtained through borehole drilling present challenges in terms of accuracy. By obtaining investigations of the entire geological formation, there is a possibility to construct a geological model that is more precise and accurate. In our research project [6], we specifically focus on outcrops, which are exposed parts of geological formations visible on the Earth’s surface and are covered by surface soil and vegetation. By analyzing images of outcrops, we aim to identify optimal locations for storage by creating high-precision geological models. Therefore, this study aims to explore the optimal backbone for semantic segmentation of outcrop images using deep learning techniques, taking into consideration both the latest advancements in the field and computational efficiency to minimize processing time.

**Figure 1:**
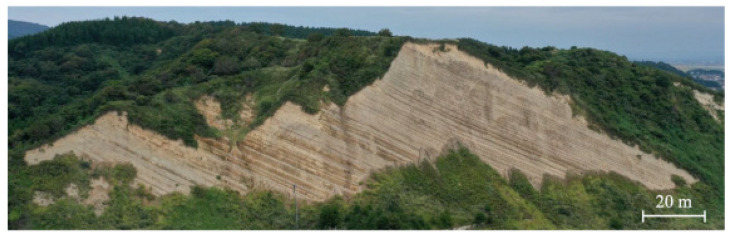
Oibanazaki Outcrop located at the southern tip of the Oga Peninsula, Akita, Japan (39${}^{\circ }$95′00″ N, 139${}^{\circ }$90′00″ E).

In this study, the primary focus was on the examination of the outcrop shown in the photograph presented in Figure 1. This paper presents our research efforts in developing a precise and efficient methodology for the classification of geological formations in outcrop images. Our approach leverages deep learning-based semantic segmentation techniques for this purpose, aiming to achieve accurate and reliable results. We investigate various backbone architectures to determine the most suitable approach for this task. By accurately characterizing geological formations, our proposed methodology can contribute to identifying optimal locations for CCS and promoting effective carbon sequestration, which is crucial for mitigating the impact of climate change.

## 2. Related Studies

The field of computer vision has extensively utilized segmentation techniques for pixel-wise object classification in images. Segmentation serves as a fundamental technology in various practical tasks, including autonomous driving and other computer vision applications [7]. Until recently, Convolutional Neural Networks (CNNs) [8] have been the predominant approach for segmentation tasks in computer vision, following the introduction of Fully Convolutional Networks (FCN) [9] and their subsequent advancements. In 2020, Vision Transformer (ViT) [10] was introduced as an architecture that adapted the successful Transformer model [11] from natural language processing to image recognition. ViT surpassed the state-of-the-art BiT (Big Transfer) [12] method in terms of accuracy, leading to a surge of research utilizing ViT in the field of image segmentation.

As a transformer-based architecture for image recognition, ViT builds upon the Transformer model that revolutionized natural language processing tasks. By omitting convolutional operations, ViT achieves improved computational efficiency and scalability. Unlike CNNs, Transformers lack the inherent inductive bias that considers the proximity of information in convolutional layers as relevant, necessitating a large amount of training data for generalization. Geirhos et al. [13] highlighted the classification characteristics of CNNs, which prioritize textures over object shapes, as differing from human perception. Conversely, Tuli et al. [14] revealed that ViT’s classification characteristics are biased towards object shapes and align more closely with human perception.

In recent years, there has been a growing interest in investigating not only CNNs and ViTs as individual backbone architectures but also hybrid backbone architectures that combine both approaches. Moreover, there has been a resurgence of interest in employing a simplistic backbone architecture that solely comprises Multi-Layer Perceptron (MLP) networks [15]. MLPs are feedforward neural networks composed of multiple layers of interconnected perceptrons, each consisting of weighted inputs, an activation function, and a bias term. MLPs can be seen as a deep extension of traditional neural network architectures, where the concept of depth refers to the increased number of layers in the network. By stacking multiple layers, MLPs can capture hierarchical representations of input data, enabling them to learn intricate and abstract features through their deep structure. This deep layering allows MLPs to effectively model complex relationships and patterns in the input data, making them powerful tools in various machine learning tasks. While MLP-based backbones demonstrate performance comparable to BiT and ViT in classification tasks, their applicability to segmentation tasks has not been fully explored.

Traditionally, high-precision 3D models of geological strata were created using ground-based laser scanning methods [16]. However, this approach has limitations such as the weight of surveying equipment, the need for scans from multiple field-based positions, and the time-consuming nature of data acquisition. Consequently, the modeling process presents significant challenges in regions where conducting in situ measurements and data collection is impractical or entails risks. In such cases, drones equipped with cameras are being utilized. Researchers such as Corradetti et al. [17] employed drones to capture photographs of cliffs composed of nearly vertical outcrops, creating 3D models that were used for crack analysis and understanding crack propagation patterns. Similarly, Sharad et al. [18] used drones to capture high-resolution images of complex and hazardous landslides, generating cm-level accuracy 3D models. Javier et al. [19] employed drones to create highly accurate and high-resolution 3D models for identifying and interpreting ancient Roman gold mining sites in Northwestern Spain, revealing areas such as excavation sites, canals, reservoirs, and drainage channels. Mirkes et al. [20] proposed a semantic segmentation method for rock outcrops that leads to the detection and segmentation of various geometric features, including fractures, faults, and sedimentary layers. Zhang et al. [21] state that most existing semantic segmentation methods are based on FCNs, which replace the fully connected layer with fully convolutional layers for pixel-level prediction. Malik et al. [22] proposed a segmentation method using a model that combines U-Net [23] and LinkNet [24] to classify three classes: background, sandstone, and mudstone. They conducted an evaluation experiment on a self-collected dataset of 102 images from a field in Brunei Darussalam, demonstrating higher accuracy compared to conventional methods. However, their proposed method and the comparison methods were based on conventional CNN-based backbones, without considering recent advancements in deep learning. Vasuki et al. [25] proposed an interactive segmentation method primarily using edge features extracted from rock images obtained using a drone. They focused on superpixels as the minimum resolution, emphasizing geological analysis and engaging in image sensing and analysis. However, their segmentation method relied on conventional image processing methods for feature extraction and did not incorporate learning-based techniques, which might not provide sufficient accuracy and generalizability for this application domain.

Although research utilizing drones in geosciences has gained momentum [26], most studies focus on analyzing topography using 3D models generated from captured photographs. However, there are a limited number of reported studies [22,25] that apply segmentation-based approaches to classify lithostratigraphy in geological outcrops. This paper emphasizes the mounting interest in employing CNNs, ViT, and investigating hybrid backbone architectures for segmentation tasks, while also acknowledging the expanding utilization of drones in geological studies. Furthermore, it identifies a research gap concerning the application of segmentation-based approaches to the lithostratigraphic classification of geological outcrops.

## 3. OutcropHyBNet

**Figure 2:**
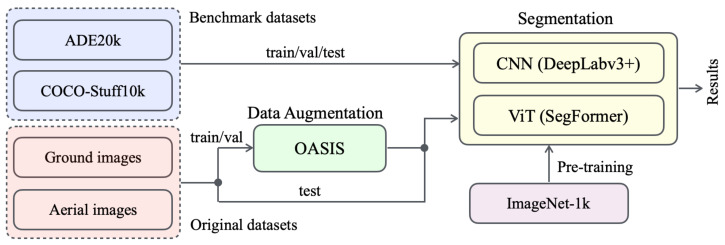
Overall architecture of OutcropHyBNet.

We propose a novel approach named OutcropHyBNet, which combines a state-of-the-art CNN architecture, DeepLabv3+ [27], and a transformer-based vision model, SegFormer, to tackle the task of stratum semantic segmentation in outcrop images. The overall architecture of our proposed method is illustrated in Figure 2. OutcropHyBNet leverages the robust segmentation capabilities of DeepLabv3+ and the expressive power of SegFormer [28] as the backbone networks for accurate and efficient stratum segmentation. To enhance the diversity of training data, we employ Only Adversarial Supervision for Semantic Image Synthesis (OASIS) [29] in image synthesis. During the segmentation training process, our dataset includes both original outcrop images and synthetic images generated using OASIS. OASIS utilizes the power of generative models to produce synthetic outcrop images that manifest characteristics closely resembling those observed in real-world data. By incorporating OASIS-generated images into the training dataset, we expand the available data and improve the model capability to handle various outcrop images.

The OutcropHyBNet architecture is designed to harness the power of CNN and ViT backbones for accurate semantic segmentation of outcrop images. The input images are processed through both backbones, allowing for efficient feature extraction and comprehensive contextual understanding. The extracted features are further processed by additional layers to perform pixel-wise classification, resulting in the generation of high-quality segmentation maps. As the baseline model for OutcropHyBNet, we integrate DeepLabv3+ and SegFormer into the architecture. Herein, SegFormer is one of the state-of-the-art semantic segmentation models that adopts a transformer-based architecture [30]. By leveraging the capabilities of SegFormer, we aim to improve the accuracy and performance of outcrop image segmentation in our proposed method. By contrast, DeepLabv3+ is a lightweight model that exhibits superiority in stuff classification. Although the ViT has gained significant attention in the field of computer vision, CNNs still demonstrate strong potential in segmentation tasks, particularly in areas involving texture and stuff [31]. For this mechanism, OutcropHyBNet can flexibly utilize both backbones based on the segmentation target.

### 3.1. Semantic Image Synthesis

#### 3.1.1. Data Augmentation with GANs

Generative Adversarial Networks (GANs) [32] are a generative model based on adversarial training without extensively annotated training data [33]. GANs offer a technique for generating realistic data, such as images, from random noise. In our previous study [34], we demonstrated the power and effectiveness of image synthesis for semantic segmentation applications in agriculture.

The network architecture of GANs consists of two main components: a generator *G* and a discriminator *D*. The *G* is responsible for generating synthetic images, while the role of *D* is to distinguish between real images from a dataset and fake images generated by *G*. The *G* aims to deceive *D* by generating images that closely resemble real ones, while *D* strives to accurately classify the input images as real or fake. Both *G* and *D* networks are trained adversarially and simultaneously. The training process involves iteratively updating the networks in an attempt to achieve a dynamic equilibrium, where *G* becomes increasingly proficient at generating realistic images, and *D* becomes increasingly adept at discriminating between real and fake images. The *G* receives random noise as input and transforms it into synthesized images. The *D*, on the other hand, receives either real images from a dataset or generated images from *G* as input and outputs a probability score indicating the likelihood of the input being real.

By optimizing the respective objectives of *G* and *D* through backpropagation and gradient descent, GANs learn to generate high-quality synthetic data that closely resembles the real data distribution. Since their introduction, GANs have undergone significant improvements and spawned various derivative models. These improvements have expanded the capabilities of GANs and paved the way for extensive research in the realm of semantic image synthesis. In this study, we introduce OASIS [29], a novel generative model based on GANs, which harnesses the power of the adversarial training paradigm to synthesize images with desired semantic content.

#### 3.1.2. OASIS

In recent years, research on data generation has gained significant momentum, driven by the introduction of diffusion models (DMs) [35]. Although DMs have demonstrated their efficacy in various vision tasks [36], they often require substantial computational resources and impose a heavy memory burden. In this study, prioritizing ease of implementation and computational efficiency, we have selected OASIS [29] as our model of choice, which is based on the GAN framework.

To generate high-quality images that align with the input semantic label map, *G* requires *D*, which can effectively capture semantic features at various resolutions. In the OASIS framework, the role of *D* is structured as a multi-class segmentation task. The architecture adopted in *D* is an encoder-decoder network, specifically based on the U-Net [23] with skip connections. The segmentation task for *D* aims to predict per-pixel class labels for real images, considering the given semantic label map as the ground truth. In addition to the *N* semantic classes obtained from the label map, all pixels of the synthesized images are classified as an additional class. Therefore, the formulated segmentation task involves N+1 classes, and OASIS employs a cross-entropy loss with N+1 classes for training.

As the segmentation task deals with class imbalance due to varying class frequencies, there is a possibility that the performance may be hindered. To mitigate this issue, OASIS leverages pixel-level loss calculation in *D*. Specifically, each class is weighted inversely proportional to the frequency of occurrence at the pixel level within a batch. This weighting scheme assigns higher weights to classes with lower frequencies, aiming to alleviate the impact of class imbalance and improve accuracy for classes with low occurrence. As a result, the contribution of each class to the loss is normalized, leading to improved accuracy for classes with low occurrence. The loss $L_{D}$ of the updated *D* is formulated as follows:

(1) $$L_{D}=-E_{\left(x,t\right)}\left[\sum_{i,j}^{H\times W}\log D{\left(G\left(z,t\right)\right)}_{i,j,c=N+1}\right]$$

where *x* represents real images, *H* and *W* represent the image height and width, (z,t) is the combination of noise and label map used by *G* to produce synthesized images, and *D* maps real or synthesized images to per-pixel (N+1)-class prediction probabilities. Here, E denotes a unit vector in a normed vector space. The ground truth label *t* is a 3D tensor, where the first two dimensions correspond to spatial positions (i,j)∈H×W, and the third dimension encodes the class c∈1,…,N+1 as a one-hot vector. When designing *G* to align with *D*, the loss function for *G* is expressed as following.

(2) $$L_{G}=-E_{\left(z,t\right)}\left[\sum_{c=1}^{N}\alpha_{c}\sum_{i,j}^{H\times W}t_{i,j,c}\log D{\left(G\left(z,t\right)\right)}_{i,j,c}\right].$$

To enable multi-modal synthesis through noise sampling, *G* is designed to synthesize diverse outputs from input noise. Hence, a noise tensor of size M×H×W is constructed to match the spatial dimensions of the N×H×W label map, where *N* represents the number of semantic classes, and *M* corresponds to the number of masks. During training, the 3D noise tensor is sampled channel-wise and fed to each pixel of the image. After sampling, the noise and label maps are concatenated along the channel dimension, forming a (M+N)×H×W noise-label concatenation 3D tensor. This concatenation tensor serves as input to the first generation layer and spatially adaptive normalization layers of each generation block. The 3D noise has sensitivity at the channel and pixel levels, allowing for specific object-level image generation by sampling noise locally for each channel, label, or pixel during testing.

### 3.2. Semantic Segmentation

#### 3.2.1. DeepLabv3+

For pixel-level image segmentation, DeepLabv3+ [27] represents a significant advancement within the renowned DeepLab model family [37]. This architecture has been specifically designed to excel in the task of precise and detailed segmentation, offering exceptional performance and accuracy. By leveraging advanced techniques and innovations, DeepLabv3+ pushes the boundaries of pixel-level image segmentation and stands as a testament to the ongoing progress within the DeepLab model family. DeepLabv3+ has garnered significant acclaim for their remarkable prowess in achieving precise and efficient semantic image segmentation. With its enhanced architecture and refined techniques, DeepLabv3+ builds upon the foundation established by its predecessors, pushing the boundaries of segmentation capabilities even further. Moreover, DeepLabv3+ has achieved outstanding performance on various benchmark datasets, surpassing previous state-of-the-art methods in terms of accuracy and computational efficiency. Its ability to capture contextual information at multiple scales and preserve fine details has made it particularly effective in tasks such as object recognition, scene understanding, and medical image analysis.

The architecture of DeepLabv3+ builds upon the strengths of its predecessors by incorporating an encoder-decoder structure along with atrous convolutions [38] and atrous spatial pyramid pooling (ASPP) modules [27]. The encoder network, usually based on pre-trained CNNs such as ResNet [39] or Xception [40], extracts high-level features from the input image while preserving spatial information. The atrous convolutions enable the network to capture multi-scale contextual information without significantly increasing the computational cost. The decoder network employs bilinear upsampling to restore the spatial resolution of the features obtained from the encoder. Additionally, skip connections from earlier layers are incorporated to ensure that fine-grained details are preserved in the final segmentation. The ASPP module further enhances the receptive field of the network by applying atrous convolutions at multiple dilation rates and capturing contextual information at different scales.

#### 3.2.2. SegFormer

SegFormer [28] adopts a ViT-based methodology, leveraging its distinctive Mix Transformer (MiT) encoder. The MiT encoder consists of a hierarchical Transformer, overlapped patch merging, efficient self-attention, and Mix-FFN. These components collectively contribute to the effectiveness and efficiency of the SegFormer model for segmentation tasks. Unlike ViT, which can only generate feature maps at a single resolution, the hierarchical Transformer in SegFormer produces multi-level feature maps. These maps provide both high-resolution coarse features and low-resolution fine-grained details, contributing to improved segmentation accuracy.

ViT incorporates Positional Encoding (PE) to capture positional information. However, the resolution of PE is fixed. As a result, when the resolution differs between training and testing, the accuracy may deteriorate. To address this issue, a Mix-FFN is introduced, which applies a 3×3 convolutional layer directly to the feed-forward network (FFN).

SegFormer adopts a lightweight decoder consisting solely of MLP layers, known as the All-MLP decoder. This avoids the computationally expensive configurations used in other methods. The hierarchical Transformer encoder in SegFormer enables this simple decoder by having a larger effective receptive field (ERF) compared to the encoder of traditional CNNs.

### 3.3. Cross-Entropy Loss

To train DeepLabv3+ and SegFormer, a large-scale dataset annotated with pixel-level labels is required. Typically, the network is trained in a supervised manner using a cross-entropy loss function $L_{CE}$ given by:

(3) $$L_{CE}=-\frac{1}{N}\sum_{i=1}^{N}\sum_{j=1}^{C}y_{ij}\log\left(p_{ij}\right),$$

where *N* is the number of pixels; *C* is the number of classes; $y_{ij}$ represents the ground truth label for pixel *i* and class *j*; and $p_{ij}$ is the predicted probability of pixel *i* belonging to class *j*.

### 3.4. Evaluation Criteria

In this study, we employ the Fréchet Inception Distance (FID) [41,42] as a formal evaluation criterion. By incorporating information about the underlying distributions and the representation of features, the FID metric provides a comprehensive assessment that captures the fidelity and resemblance of the generated samples to the real data. The FID metric utilizes a pre-trained Inception network [43] that has been trained on the ImageNet dataset [44]. The pre-training on ImageNet helps capture general visual features and enables transfer learning, where the learned representations are fine-tuned for specific tasks [45]. By leveraging the representation power of the Inception network, FID provides a quantitative measure of the quality and diversity of generated images compared to the real image distribution. FID calculates the distance between the feature vectors extracted from the real images and the generated images, quantitatively evaluating the similarity between the two. The FID is defined as follows:

(4) $$\mathrm{FID}={∥m-m_{w}∥}^{2}+Tr\left(C+C_{w}-2\sqrt{C_{w}}\right),$$

where $m_{w}$ and *m* represent the means of the feature vectors extracted from the generated images and the real images, respectively. $C_{w}$ and *C* represent the covariance matrices of the feature vectors.

Subsequently, to assess the quality of segmentation, Intersection over Union (IoU) is employed as the evaluation metric in this study. IoU represents the degree of intersection between the predicted region and the ground truth region, and mIoU represents the average IoU across all classes. IoU is calculated using the following equation:

(5) $$\mathrm{IoU}=\frac{TP}{TP+FP+FN}.$$

Herein, True Positive (TP) corresponds to the instances where both the prediction and the class are true. False Positive (FP) represents the instances where the prediction is false, but the class is true. False Negative (FN) denotes the instances where the prediction is true, but the class is false.

## 4. Preliminary Performance Evaluation with Benchmark Datasets

### 4.1. Data Profiles and Setups

We evaluated the performance of the proposed method, OutcropHyBNet, in a general context using two benchmark datasets: COCO-Stuff10K [46] and ADE20K [47]. These datasets encompass diverse scenes and objects commonly encountered in everyday environments, facilitating a comprehensive evaluation of the proposed method’s performance in real-world scenarios.

The COCO-Stuff10K serves as an extensively utilized benchmark dataset for tasks related to scene understanding and segmentation. Comprising 10,000 high-resolution images, this dataset features pixel-wise annotations. The images within this dataset exhibit diverse resolutions, ranging from 480×640 to 960×1280 pixels, while maintaining an aspect ratio of 3:4. The dataset provides comprehensive annotations for both objects and stuff categories. It includes 80 object categories, such as person, car, and dog, and 91 stuff categories, such as sky, grass, and road. The pixel-level annotations enable detailed semantic segmentation of scenes, facilitating the evaluation and development of advanced computer vision algorithms. Moreover, the dataset provides a wide array of visual scenes, encompassing a comprehensive spectrum of both indoor and outdoor environments. It serves as a standard benchmark for evaluating and contrasting the performance of semantic segmentation models.

The ADE20K dataset is a widely used dataset for semantic segmentation tasks. It comprises more than 20,000 high-resolution images, specifically 150 objects and 50 stuff categories. All images in the dataset have a fixed resolution of 512 × 512 pixels. The ADE20K dataset provides pixel-level annotations for both objects and stuff categories, enabling fine-grained semantic segmentation. It covers a diverse range of scenes, including indoor and outdoor environments, and captures various objects and stuff categories commonly encountered in everyday life. Moreover, the ADE20K dataset is designed to facilitate research and development in scene parsing and semantic understanding. It serves as a benchmark for evaluating the performance of semantic segmentation models and has been widely adopted in the computer vision community. The inclusion of this dataset allows for a comprehensive assessment of the generalization capability of the proposed method, OutcropHyBNet.

### 4.2. Experimental Setup

For this study, we utilized MMSegmentation [48], an open-source segmentation toolbox developed by OpenMMLab, as the designated implementation platform. MMSegmentation offers a comprehensive and versatile solution specifically tailored for semantic segmentation tasks. Its open-source nature and seamless integration with PyTorch provide us with a valuable resource for conducting our evaluation experiments. One of the key strengths of MMSegmentation lies in its wide array of segmentation models, catering to diverse requirements in the field. This rich collection of models establishes MMSegmentation as an invaluable asset for various developers. With its extensive toolkit, we can effectively address various segmentation tasks and explore different approaches, thereby enhancing the depth and breadth of our research and practical applications.

The computation for this study was carried out on a single NVIDIA RTX A6000 GPU. Renowned as a high-performance GPU, the A6000 is purpose-built to tackle professional workloads in various fields, including data science, deep learning, AI research, and content creation. Its exceptional capabilities make it an ideal choice for handling the intensive computational tasks. The A6000 has 10752 CUDA cores, 48 GB of GDDR6 memory, and a memory bandwidth of 768 GB/s. With its powerful architecture, it delivers exceptional performance for tasks such as deep learning training, real-time ray tracing, and high-resolution rendering. The parameters for each method were determined using the configuration file of the pretrained model that achieved the highest accuracy on the ADE20K dataset, which is provided by MMSegmentation [49].

### 4.3. Class Balancing for Uneven Data

To mitigate the challenge of class imbalance [50], we employed class balancing techniques [51] as a simple and practical approach for data adjustment and enhancement. Let *x* represent the number of pixels in a class and *y* represent the total number of pixels excluding unlabeled pixels. The weight *w* is calculated using the following equations:

(6) $$z=\frac{x}{y}\;\mathrm{and}\;w=\frac{\bar{z}}{z}$$

where $\bar{z}$ represents the median of *z*.

Table 1 provides a comprehensive overview of the calculated weights, which were determined considering the pixel occupancy ratio. The weights were assigned in such a way that they decrease as the pixel occupancy ratio increases, and conversely, they increase as the pixel occupancy ratio decreases. The approach aims to effectively mitigate the issue of class imbalance by assigning higher weights to underrepresented classes with lower pixel occupancy ratios. This strategy ensures that these classes receive greater attention during the training process, thereby addressing their significance in a more comprehensive manner. By incorporating these calculated weights, we aim to achieve a more balanced and accurate model performance, ultimately improving the overall effectiveness of our approach in handling imbalanced datasets.

We evaluate the performance of the models using the Intersection over Union (IoU) metric for each class. Table 2 presents the comparison of class balancing results for both DeepLabv3+ and SegFormer models. From the results, we observe that class balancing has a significant impact on the performance of both models. SegFormer shows improvements in most classes, except for the Black class. The decrease in performance for the Black class in SegFormer can be attributed to a specific image (Image 10), where the IoU is significantly lower compared to other images. The IoU for the Black class in DeepLabv3+ remains relatively stable across all images.

### 4.4. Data Augmentation

Our proposed approach, OutcropHyBNet, utilizes OASIS to generate images and augment the dataset. By leveraging OASIS-generated images, we expand the breadth and depth of our dataset, enabling a more comprehensive representation of geological features and variations. Integrating OASIS into our methodology addresses the challenge of limited real-world outcrop data and enriches the learning process of OutcropHyBNet. The combination of synthetic and real data enhances the model’s capacity to accurately analyze and interpret geological formations with improved precision and reliability.

Table 3 presents the parameters used for this purpose. In this experiment, a dataset with a sampling number of 256 images was utilized, and the same dataset was used for both training and testing. DeepLabv3+ and SegFormer were used as the comparative methods. A total of 3661 images were used for evaluation, which consisted of 333 images generated using OASIS and 256×13 images from the dataset used in the full image experiment. The training and testing data were randomly allocated in a 9:1 ratio, resulting in 3294 images for training and 367 images for testing.

Table 4 shows the results of class balancing. For the Black class, both methods improved accuracy in 4 out of 6 images. For the Red class, DeepLabv3+ improved accuracy in 10 out of 13 images, while SegFormer improved accuracy in 8 images. Similarly, for the Cyan class, DeepLabv3+ improved accuracy in 11 out of 13 images, and SegFormer improved accuracy in 8 images. Regarding the Yellow class, DeepLabv3+ improved accuracy in 6 out of 13 images, while SegFormer improved accuracy in 4 images. In terms of mIoU, DeepLabv3+ improved accuracy in 10 out of 13 images, and SegFormer improved accuracy in 6 images. It is worth noting that the Yellow class exhibited a decrease in accuracy in more than half of the images for both methods. This can be attributed to the initial weight of 0.3792, which is significantly lower compared to the absence of class balancing.

Table 5 demonstrates the improved mIoU scores achieved by incorporating OASIS-generated images. These images significantly enhance the accuracy of segmenting and classifying geological formations in our proposed approach. Both segmentation methods showed improved accuracy, denoted Δ, for all classes compared to the dataset before augmentation. Particularly, they achieved an accuracy improvement of over 3% for the Cyan class. Therefore, it can be concluded that dataset augmentation using OASIS for data generation contributes to the improvement in accuracy. Furthermore, the consistent trend of CNNs backbones outperforming ViT backbones was observed throughout the evaluation.

### 4.5. Selection of Backbones

To verify the effectiveness of the proposed approach, a preliminary experiment was conducted for performance comparison using seven different network models with varying backbones. The backbones used for comparison were ResNet [39], HRNet [52], U-Net [23], Swin Transformer [53], MiT [28], ViT [10], and SVT [54]. Table 6 presents the specific parameter configurations for each backbone utilized in this experiment. The common parameters included a batch size of 8, a class count of 4, 4 sampling patterns (64, 128, 256, and 512 images), an input image size of 256 × 256 pixels, and a training epoch set to 50. Regarding the input data, a random sampling was performed on 13 images, allocating them to training and testing data in a 9:1 ratio.

**Figure 3:**
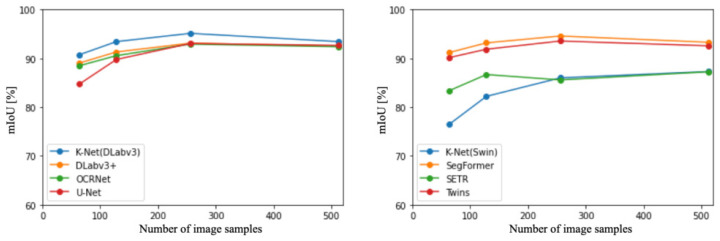
Comparison of CNN-based and ViT-based methods in terms of accuracy at different sampling numbers.

The left panel of Figure 3 illustrates the accuracy of CNN-based methods [37]. DeepLa-bv3+ [27] consistently demonstrated the highest accuracy among all sampling numbers. Additionally, across all methods, the highest accuracy was achieved when the sampling number was 256 images. Subsequently, the right panel of Figure 3 presents the accuracy of ViT and hybrid-based methods [28,57]. SegFormer consistently exhibited the highest accuracy across all sampling numbers. Moreover, excluding SEgmentation TRansformer (SETR) [57], SegFormer achieved the highest accuracy when the sampling number was 256 images.

**Figure 4:**
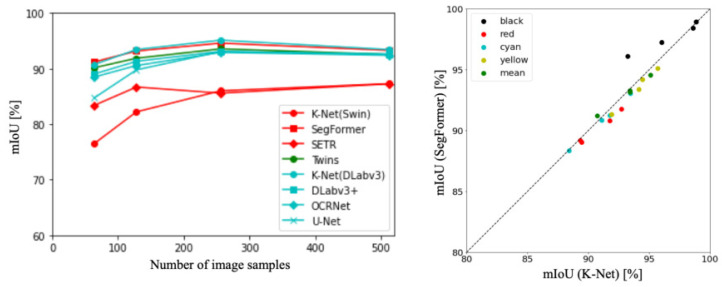
Accuracy trends and distributions for all backbones and top two models.

Figure 4 depicts the accuracy trends and distributions for all backbones and the top two models. The red lines correspond to ViT-based backbone [10], the green lines represent hybrid backbones [57], and the blue lines represent CNN-based backbones [38]. The graph visually depicts how the accuracy of these methods varies across different experimental settings. Comparing the results, the methods can be ranked in terms of accuracy as follows: DeepLabv3+ [27], SegFormer [28], Twins [54], ResNet [39], and ViT [10]. In other words, on the original dataset, CNNs outperformed ViT in terms of accuracy for this context.

Table 7 presents mIoU of each class [27,28]. Comparing the results, DeepLabv3+ demonstrated superiority for all classes except for the Black class at sampling numbers of 64. Additionally, in all sampling numbers except for 64 images, DeepLabv3+ outperformed SegFormer. Analyzing the mean scores, DeepLabv3+ consistently showed superior performance in all classes. Furthermore, in both methods, the classes with the highest accuracy were ranked as follows: Black, Yellow, Cyan, and Red.

### 4.6. Segmentation Results

**Figure 5:**
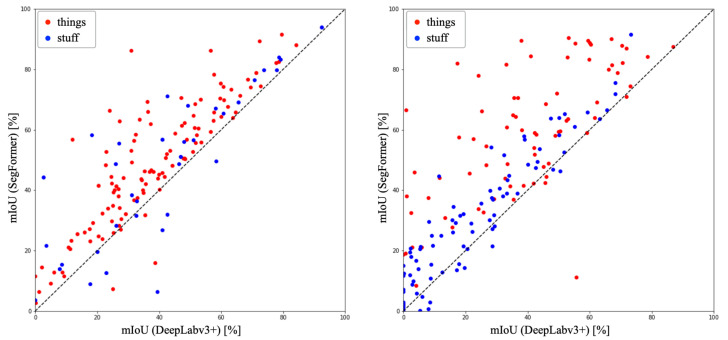
Scatter plots of accuracy for DeepLabv3+ and SegFormer.

Figure 5 shows the comparison results for all classes in each dataset. In ADE20K, DeepLabv3+ achieved an mIoU of 29.36%, while SegFormer achieved an mIoU of 41.38%. SegFormer demonstrated superiority in 154 out of 171 classes (90% of the total classes). In COCO-Stuff10K, DeepLabv3+ achieved an mIoU of 38.78%, while SegFormer achieved an mIoU of 48.40%. SegFormer exhibited superiority in 138 out of 150 classes (92% of the total classes, see Appendix A).

In COCO-Stuff10K, DeepLabv3+ outperformed SegFormer in terms of accuracy for certain classes. Among the things classes, DeepLabv3+ exhibited higher accuracy than SegFormer in four classes: surfboard, sports ball, car, and mouse, out of the 80 classes. In the stuff classes, DeepLabv3+ demonstrated higher accuracy in 13 classes: platform, mountain, stone, straw, bush, bridge, roof, house, cabinet, floor-other, float-wood, carpet, and wall-panel, out of the 91 classes. Conversely, SegFormer showed higher overall accuracy compared to DeepLabv3+ in both datasets. Similarly, in ADE20K, DeepLabv3+ surpassed SegFormer in accuracy for specific classes. Among the things classes, DeepLabv3+ achieved higher accuracy than SegFormer in 4 classes: railing, base, food, and monitor, out of the 115 classes. In the stuff classes, DeepLabv3+ demonstrated higher accuracy in 8 classes: house, river, skyscraper, hovel, path, tower, stairway, and pier, out of the 35 classes. Once again, SegFormer exhibited higher overall accuracy than DeepLabv3+ in ADE20K. In both datasets, the percentage of classes where DeepLabv3+ showed superiority was higher in the stuff classes compared to the things classes. This can be attributed to the fact that stuff classes lack well-defined boundaries, and the CNN-based architecture of DeepLabv3 utilized by DeepLabv3+ may have provided an advantage in texture classification, as mentioned earlier.

Table 8 presents the top score classes observed in the COCO-Stuff10K dataset, while Table 9 showcases the top score classes identified in the ADE20K dataset. These tables provide a comprehensive overview of the most prominent classes present in each dataset, shedding light on the prevalent semantic categories and objects captured in the respective datasets. The identification and analysis of these top score classes contribute to a deeper understanding of the dataset composition and can inform the development of more effective models and algorithms for semantic segmentation and scene understanding tasks.

Focusing on the stuff classes, which are the classes of interest in this study, the top 10 classes combined for both methods include 3 classes (15%) in COCO-Stuff10K and 8 classes (40%) in ADE20K. On the other hand, the bottom 10 classes combined include 24 classes (75%) in COCO-Stuff10K and 9 classes (45%) in ADE20K. Therefore, it can be inferred that stuff classes have a lower representation in the top classes and a higher representation in the bottom classes.

## 5. Outcrop Segmentation

### 5.1. Custom Dataset Profile

To assess the effectiveness of the proposed method, we employed two custom benchmark datasets: stationary camera-captured ground-level images and aerial images captured by drones. The stationary images dataset consists of a collection of images captured from the perspective of a person on the ground, with meticulous attention given to their inclusion and additional insights provided by domain experts. These images were taken using a Ricoh GR III camera, an off-the-shelf device widely recognized for its high-quality imaging capabilities.

The aerial images dataset comprises images captured from drones flying at varying altitudes. These images afford a bird’s-eye view perspective, facilitating the analysis of expansive scenes and the capture of distinctive visual information. The dataset includes diverse landscapes, urban areas, and natural environments, enabling the evaluation of the proposed method’s effectiveness in aerial image analysis tasks. Both datasets were carefully curated and annotated to provide ground truth labels for evaluation. The inclusion of these custom evaluation datasets allows for a thorough assessment of the proposed method’s performance across different viewing angles and environments.

#### 5.1.1. Stationary Ground-Level Images

**Figure 6:**
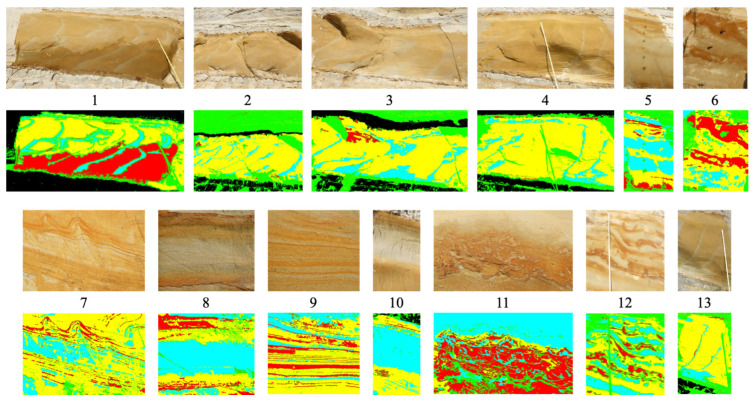
Original and annotation images of our custom dataset.

Figure 6 presents the original images from our dataset, accompanied by their corresponding annotation images. The images were annotated by geological experts, who selectively cropped them to capture the regions of interest (RoI). Consequently, the image sizes exhibit variability due to the purposeful RoI extraction limited to the pertinent areas.

Table 10 presents the relationship between geological lithology, grain size, grain sorting, and annotation colors: Yellow, Cyan, Red, and Black. Average grain size is shown on the Krumbein ϕ scale based on geological analysis. The degree of grain sorting depends on the particle size classification.

Table 11 presents the resolutions in each image. Due to the burden of annotation, we clipped saliency partical images as RoIs because this is the standard way of annotation by geological experts. The burden is extremely high if the full sizes of images are set to annotation targets.

Table 12 presents the pixel frequency for each class, providing a comprehensive overview of the distribution of pixels among different semantic classes. The presence of class imbalance within the dataset necessitates the implementation of class balancing techniques to ensure equitable representation and promote accurate model performance.

Our custom dataset comprises outcrop images captured using a stationary camera. These images were manually annotated by domain experts specializing in geological analysis, using four labels. For the sake of convenience, unlabeled regions were assigned None, represented by the Green label. This labeling approach facilitates the handling of regions without specific semantic attributes. The semantic classes were allocated using a color scheme, with the unlabeled pixels represented by the color green, and the labeled pixels distributed among Black, Red, Cyan, and Yellow, resulting in a total of four labels used for classification. The green pixels were excluded from the calculations, and thus the classification was performed using the remaining 4 labels across a total of 13 images, as shown in Figure 6.

#### 5.1.2. Aerial Images

**Figure 7:**
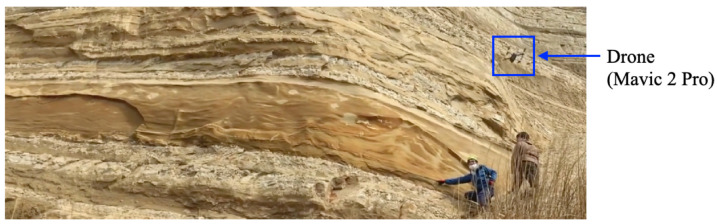
Process of capturing aerial images with the involvement of geological experts and a drone.

We have utilized various types of drones for sensing the vertical distributions of CO${}_{2}$ [58], horizontal distributions of particulate matter [59], and crops in rice paddy fields [60]. For this study, aerial images were obtained using the DJI Mavic 2 Pro, which is a compact drone manufactured by DJI. The process of capturing the images is depicted in Figure 7. The scale of the outcrop can be visually compared with the size of the two individuals captured in the photograph.

**Figure 8:**
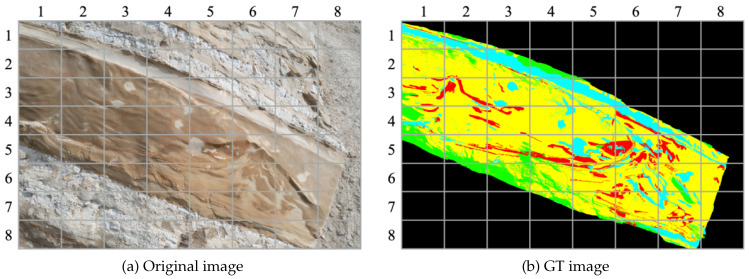
Selected aerial image for evaluation.

Among the collected images, one specific image was chosen for evaluation, as depicted in Figure 8a. We divided this image into 64 equal-sized rectangles to make it suitable for segmentation. To facilitate the evaluation process, geological experts provided annotations for five specific labels on the image, as illustrated in Figure 8b.

The image used for evaluation had a resolution of 5464×3640 pixels. The annotation data applied to this image followed the same criteria as the original dataset, performed by domain experts. The trained models used for inference were trained using the OASIS extended dataset for DeepLabv3+ and SegFormer. During inference, the aerial image was divided into an 8 × 8 grid and each sub-image was used as input. Consequently, the input size for each sub-image was 683×455 pixels. The dataset consisted of a total of 64 sub-images resulting from the division.

Table 13 presents the class-wise Intersection over Union (IoU) of each image. The scores are arranged in descending order of mIoU. Although there are 13 images, there is a 2-fold difference in accuracy. Additionally, “–” indicates images that do not contain the Black label. While the individual class-wise IoU for Black is high, it represents the average value across four images. The Red class has the lowest IoU.

Table 14 shows the correlation coefficients between the pixel occupancy ratio and ranking of each image. Note that “green” is not included as it does not affect the accuracy. The ranking of mIoU is based on the accuracy order of the average mIoU for both methods. The correlation coefficient represents the correlation between the ranking of the class’s pixel occupancy ratio and the ranking of mIoU. Negative correlation was observed for “red”. This can be attributed to the low overall pixel occupancy ratio of the Red dataset, which is 13.12%. As the pixel occupancy ratio of Red in the test data increases, the pixel occupancy ratio of Red in the training data decreases, leading to a decrease in accuracy due to insufficient data.

Negative correlation was also observed for “Cyan”, which is believed to be for the same reasons as red. On the other hand, strong positive correlation was observed for “Yellow”. This is because the overall pixel occupancy ratio of the Yellow dataset is high at 45.22%, and the training data is sufficient. Therefore, as the pixel occupancy ratio of Yellow in the test data increases, the accuracy improves. No correlation was found for “Black”. Hence, it can be inferred that the imbalance in pixel occupancy ratio affects the accuracy. This is likely due to data insufficiency, indicating the need for techniques such as data augmentation to balance the pixel occupancy ratios.

**Figure 9:**
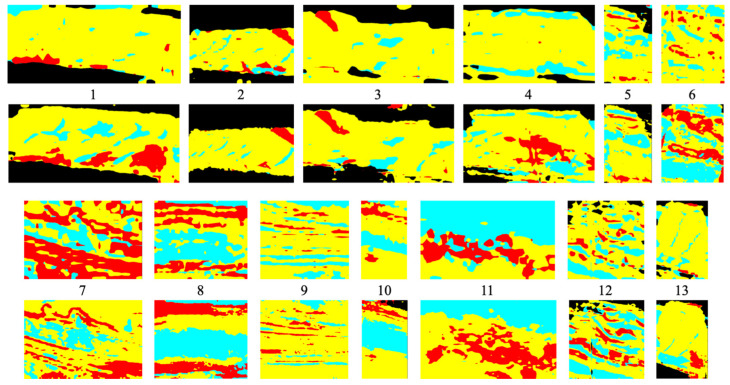
Segmentation results with DeepLabv3+ (first and third rows) and SegFormer (second and fourth rows).

Figure 9 illustrates the segmentation results. Table 15 presents the compared mIoU for each class. In terms of IoU, SegFormer demonstrates superiority across all classes. The overall IoU shows a difference of 8.18%. The largest accuracy difference is observed for the Black class, while the smallest difference is observed for the Yellow class.

**Figure 10:**
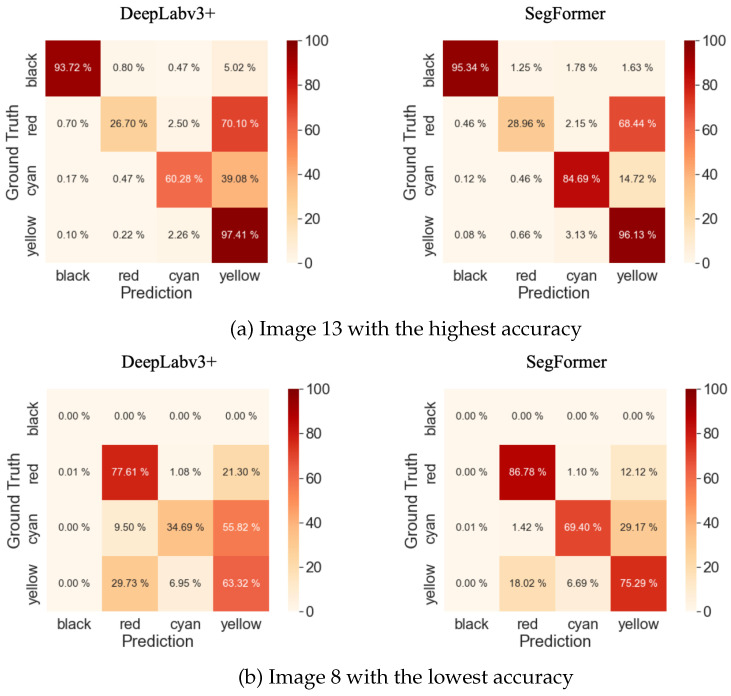
Confusion matrices of the highest and lowest accuracies.

Confusion matrices are widely used in deep learning for evaluating the performance of classification models [61]. Due to significant variations in accuracy across images, we present the confusion matrices for image 13, which has the highest accuracy, and image 8, which has the lowest accuracy, in Figure 10, as depicted in Table 14. The Confusion Matrix reveals that the accuracy of SegFormer, compared to DeepLabv3+, is 40% higher for the Black class and 16% higher for the Cyan class. This difference in accuracy contributes to the discrepancy in mIoU.

These results unequivocally demonstrate the superior performance of SegFormer in semantic segmentation compared to DeepLabv3+. The higher IoU scores obtained by SegFormer indicate its capability to better capture object boundaries and classify pixels accurately. This can be attributed to the architecture of SegFormer, which incorporates Transformer-based models, allowing for more effective feature extraction and contextual understanding. The significant accuracy difference observed for the Black class suggests that SegFormer excels in segmenting objects with complex shapes and intricate details. The Black class objects may possess fine textures or indistinct boundaries, and SegFormer’s capability to capture such nuances contributes to its superior performance. On the other hand, the minimal difference in accuracy for the Yellow class implies that both models perform similarly in segmenting objects of this class, which may have more distinguishable features or simpler shapes.

### 5.2. Segmentation Results of Aerial Images

In order to broaden the scope of validation and explore new possibilities, we applied OutcropHyBNet to aerial images for segmentation, expanding the range of applications in CCS. The segmentation results are evaluated using the mIoU metric, which assesses the accuracy and consistency of the predicted segmentation masks with respect to the ground truth masks. We applied our model, OutcropHyBNet, which had been trained using ground-level stationary images, to the aerial images in the dataset and obtain segmentation results. The model assigned a semantic label to each pixel, effectively distinguishing and categorizing different objects and regions within the image. The resulting segmented images provide a visual representation of the distinct entities present in the aerial scenes. By presenting the segmentation results obtained using OutcropHyBNet, we aim to demonstrate its effectiveness in segmenting aerial images.

**Figure 11:**
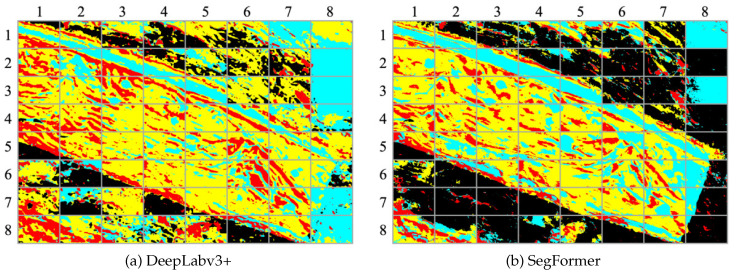
Segmentation results obtained from both backbone networks for aerial images.

Figure 11 presents the segmentation results obtained by applying DeepLabv3+ and SegFormer to the input image depicted in Figure 8a. The comparison reveals that SegFormer surpasses DeepLabv3+ in effectively capturing fine details and accurately delineating object boundaries. Specifically, a notable distinction can be observed in the segmentation results of the Black class, where SegFormer exhibits significantly improved performance compared to DeepLabv3+.

**Figure 12:**
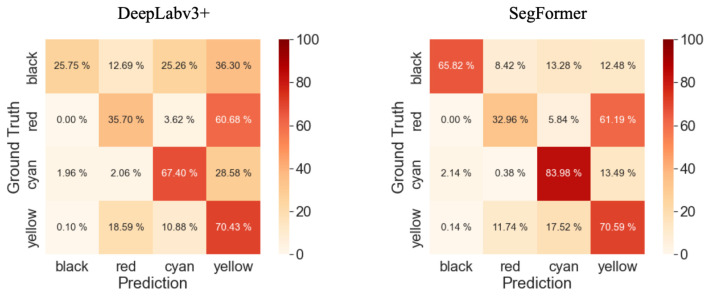
Confusion matrices for both segmentation results.

A comparison of the confusion matrices shown in Figure 12 reveals notable differences in accuracy between the two methods. Specifically, SegFormer achieves a 40% higher accuracy for the Black class and a 16% higher accuracy for the Cyan class compared to DeepLabv3+. These differences in accuracy directly contribute to the observed discrepancy in mean IoU (mIoU) between the two methods. The segmentation results produced by SegFormer exhibit clearer and more accurate delineation of the object classes, particularly for the Black and Cyan classes. On the other hand, DeepLabv3+ tends to produce more fragmented and less precise segmentation outputs. Overall, these figures visually demonstrate the superior performance of SegFormer in terms of accurate and detailed semantic segmentation compared to DeepLabv3+.

Table 16 presents the average IoU for each class. It is noteworthy that SegFormer exhibits superior performance in terms of IoU for all classes when compared to DeepLabv3+. Particularly, there is a significant 40% difference in the Black class, which results in a notable 16% difference in mIoU between the two methods. Nevertheless, the mIoU scores for both methods are below 50%, highlighting the need for further improvements to enhance the segmentation accuracy for this aerial image dataset. Examining the results in Table 16, SegFormer consistently outperforms DeepLabv3+ in capturing the fine details and boundaries of the objects, leading to higher IoU scores. The Black class exhibits the largest disparity, highlighting the difficulty of accurately segmenting this class with DeepLabv3+. On the other hand, SegFormer achieves significantly better results for the Black class, indicating its effectiveness in handling such challenging scenarios. Overall, the results demonstrate that SegFormer provides improved performance in semantic segmentation tasks, especially in capturing detailed structures and enhancing the accuracy of challenging classes.

**Figure 13:**
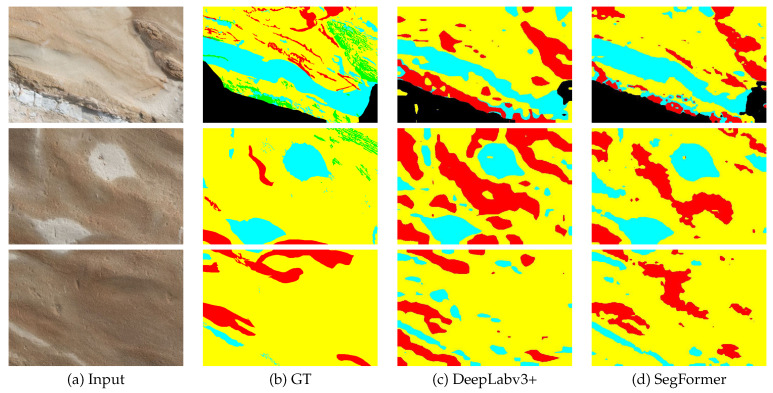
Segmentation results of the top three images.

Figure 13 illustrates the segmentation results for the top three images based on the average mIoU scores of both backbone networks on OutcropHyBNet. These images predominantly capture the central regions of the scene. This suggests that the models have successfully captured the patterns and can generalize well to unknown images. On the other hand, the bottom images predominantly contain only the outer regions with the Black class. This indicates a potential deviation in the characteristics of the Black class compared to the original dataset. To address this issue, some of the images underwent re-annotation by experts.

Table 17 presents the mIoU results after re-annotation. The fourth and sixth columns denote the differences Δ in comparison to the results obtained from the initial annotation, illustrating the changes resulting from the re-annotation process. In all conditions except for SegFormer in the 1st row and 7th column, clear improvements in accuracy are observed after re-annotation. For SegFormer in the 1st row and 7th column, the model predicted the regions that turned from Cyan to Black after re-annotation as Black, resulting in a slight improvement of less than 1% in accuracy. It can be concluded that the performance improvement was limited in this case. These results suggest the potential of deep learning models to suggest re-evaluation of annotations by humans, as they can contribute to the improvement of accuracy in semantic segmentation tasks.

**Figure 14:**
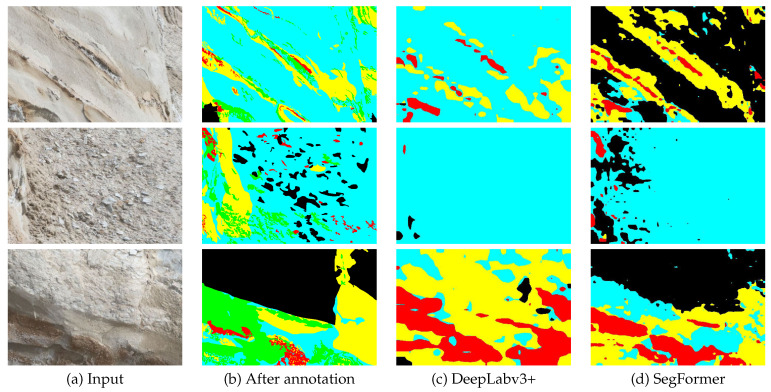
Segmentation results of three images after re-annotation.

Figure 14 presents the segmentation results of three images from Table 17 after the re-annotation process. In comparison to the results depicted in Figure 11, the colored labels in Figure 14 have been mapped according to the texture in respective stratums.

## 6. Conclusions

The objective of this study was to analyze the distribution of geological strata through the application of segmentation techniques on geological outcrop images, facilitating a comprehensive understanding of their spatial arrangement. We proposed OutcropHyBNet, which leverage DeepLabv3+ and SegFormer for semantic segmentation, along with OASIS for data augmentation. We conducted evaluations and comparisons of the classification performance and accuracy of both models across different classes using two publicly available benchmark datasets. In our preliminary experiments, we presented compelling evidence of the enhanced performance of DeepLabv3+ in classes heavily reliant on textures, particularly in the context of stuff classes. The superiority of DeepLabv3+ in accurately classifying textures within the dataset was observed to a significant extent, substantiating its effectiveness in such scenarios. In the evaluation experiments using our original datasets, we revealed that for non-standard objects with ambiguous shapes resembling geological strata, where classification depended on texture, CNNs exhibited superiority. Our study revealed that SegFormer outperformed other models in scenarios with limited data availability. Additionally, we identified that imbalanced class distributions had a notable impact on the accuracy of the models. Notably, we found that employing class balancing techniques resulted in enhanced accuracy for DeepLabv3+ compared to SegFormer. Moreover, our findings revealed that the utilization of OASIS for data augmentation significantly contributed to enhanced accuracy. By incorporating OASIS into the training process, we observed improved precision and performance in the classification task, highlighting the effectiveness of data augmentation techniques in enhancing the overall accuracy of the models. In the evaluation experiments conducted on ground-level images obtained using a stationary camera and aerial images obtained using a drone, we successfully demonstrated the superior performance of SegFormer across all classes. The comprehensive analysis revealed that SegFormer consistently outperformed other models in accurately classifying various objects and features present in the aerial images, highlighting its effectiveness and superiority in this specific context.

Our future endeavors encompass several challenges, including the augmentation of diversity through the collection of aerial images from various sources and types. By expanding our dataset to include a broader range of aerial images, we aim to improve the robustness and generalization capabilities of our models. Additionally, we plan to further explore and enhance data augmentation techniques to augment the diversity within the existing dataset, thereby fostering more comprehensive and representative training samples. Moreover, we will explore methods to improve the reproducibility of texture and color in image generation using GANs and DMs. We will also propose annotation modifications based on the inference results to further improve accuracy.

## Figures and Tables

**Table 1:** Calculated weights based on pixel occupancy ratio for class balancing.

Class	Black	Red	Cyan	Yellow
Calculated weights	1.0000	1.2570	0.7384	0.3792

**Table 2:** Comparison of class balancing results for both models [%].

Class	DeepLabv3+	Difference	SegFormer	Difference
Black	80.31	2.96	80.64	−6.85
Red	26.23	4.60	36.10	0.82
Cyan	49.35	4.81	56.08	1.57
Yellow	59.42	0.25	66.84	2.38
mean	45.64	2.97	51.19	0.35

**Table 3:** Parameters for OASIS.

Parameter	Value
Training Iterations	37,399 (200 epochs)
Generator Learning Rate	0.0001
Discriminator Learning Rate	0.0004
Batch Size	16
Input Size [pixels]	256×256
Number of Training Data	2995
Number of Testing Data	333

**Table 4:** Comparison of class balancing results [%].

Image Index	Model	Black	Red	Cyan	Yellow	Mean
1	DeepLabv3+	89.08	5.39	52.20	46.09	48.19
	SegFormer	92.18	14.36	69.47	92.80	67.20
2	DeepLabv3+	78.14	12.02	34.91	84.88	52.49
	SegFormer	64.60	19.09	31.71	85.58	50.24
3	DeepLabv3+	94.99	5.76	52.30	83.08	59.03
	SegFormer	96.35	8.75	59.20	84.04	62.09
4	DeepLabv3+	95.71	3.53	48.22	86.09	58.39
	SegFormer	95.94	10.05	53.74	91.30	62.76
5	DeepLabv3+	–	16.81	50.46	57.14	31.10
	SegFormer	–	45.32	42.81	56.62	36.19
6	DeepLabv3+	–	54.92	26.19	56.15	34.32
	SegFormer	–	61.51	26.55	54.52	35.65
7	DeepLabv3+	–	31.12	50.21	61.31	47.54
	SegFormer	–	39.67	59.78	67.43	55.63
8	DeepLabv3+	–	31.94	45.46	33.06	27.61
	SegFormer	–	42.34	71.59	45.88	39.95
9	DeepLabv3+	–	40.38	47.75	59.20	49.11
	SegFormer	–	48.87	50.59	65.66	55.04
10	DeepLabv3+	31.54	21.71	32.80	35.00	30.26
	SegFormer	40.75	30.37	49.43	41.15	40.42
11	DeepLabv3+	–	29.29	74.77	14.37	39.48
	SegFormer	–	54.02	76.17	21.85	38.01
12	DeepLabv3+	–	69.86	66.88	63.95	50.17
	SegFormer	–	78.45	69.02	69.04	54.13
13	DeepLabv3+	92.42	18.31	59.46	92.20	65.60
	SegFormer	94.02	16.50	68.96	92.99	68.12

**Table 5:** Improved mIoU [%] for OASIS Evaluation.

Class	DeepLabv3+	Δ	SegFormer	Δ
Black	98.67	1.98	98.34	0.66
Red	92.60	1.78	92.04	1.84
Cyan	94.75	3.57	94.27	3.40
Yellow	96.01	1.96	95.41	1.91
mean	95.51	2.33	95.02	1.96

**Table 6:** Model configurations for semantic segmentation.

Method	Backbone	Crop Size	Learning Rate	Weight Decay
DeepLabv3+ [27]	ResNet-101	512×512	$1.0\times 10^{-2}$	$5.0\times 10^{-4}$
OCRNet [55]	HRNetV2p-W48	512×512	$1.0\times 10^{-2}$	$5.0\times 10^{-4}$
U-Net	U-Net	512×1024	$1.0\times 10^{-2}$	$5.0\times 10^{-4}$
K-Net(Swin) [53,56]	Swin-L	640×640	$6.0\times 10^{-5}$	$5.0\times 10^{-4}$
SETR [57]	ViT-L	512×512	$1.0\times 10^{-3}$	0.0
Twins [54]	SVT-L	512×512	$6.0\times 10^{-5}$	$1.0\times 10^{-2}$
SegFormer	MiT-B5	640×640	$6.0\times 10^{-5}$	$1.0\times 10^{-2}$

**Table 7:** mIoU of each class [%].

Class	DeepLabv3+	SegFormer
Black	96.69	97.68
Red	90.82	90.20
Cyan	91.18	90.87
Yellow	94.05	93.50
mean	93.18	93.06

**Table 8:** Top score classes in COCO-Stuff10K [%].

Pixel Frequency			DeepLabv3+			SegFormer		
**Rank**	**Class**	**Ratio**	**Rank**	**Class**	**IoU**	**Rank**	**Class**	**IoU**
1	person	8.94	1	zebra	86.91	1	snow	91.54
2	tree	5.26	2	person	78.55	2	cow	90.44
3	sky-other	4.93	3	snow	73.16	3	broccoli	90.13
4	wall-other	4.80	4	stop sign	73.00	4	parking meter	89.54
5	grass	3.91	5	horse	71.81	5	bear	89.50
6	clouds	3.36	6	surfboard	71.80	6	elephant	88.81
7	building-other	2.76	7	bus	70.55	7	cat	88.63
8	dining table	2.44	8	fire hydrant	70.29	8	train	88.32
9	road	2.39	9	airplane	68.95	9	fire hydrant	87.96
10	sea	2.08	10	tree	68.23	10	zebra	87.57

**Table 9:** Top score classes in ADE20K [%].

Pixel Frequency			DeepLabv3+			SegFormer		
**Rank**	**Class**	**Ratio**	**Rank**	**Class**	**IoU**	**Rank**	**Class**	**IoU**
1	wall	15.53	1	sky	92.35	1	sky	93.98
2	building	10.56	2	bed	84.09	2	pool table	91.59
3	sky	8.65	3	pool table	79.52	3	tent	89.36
4	floor	6.11	4	road	79.11	4	bed	88.08
5	tree	4.72	5	ceiling	78.69	5	bus	86.24
6	ceiling	4.43	6	car	78.68	6	microwave	86.21
7	road	3.92	7	building	77.96	7	ceiling	83.96
8	bed	2.28	8	toilet	77.77	8	road	83.36
9	windowpane	1.95	9	floor	73.83	9	car	82.59
10	grass	1.80	10	cradle	72.71	10	toilet	82.28

**Table 10:** The relationship between geological lithology, grain size, grain sorting, and annotation colors.

Annotations	Geological Lithofacies	Average Grain Size (ϕ)	Grain Sorting
Yellow	Medium to fine sandstone	2.4	Well
Cyan	Fine to silty sandstone	2.6	Moderate
Red	Coarse to silty sandstone	3.0	Poor
Black	Siltstone	greater than 4.0	Very poor

**Table 11:** Image size and number.

Index	Resolution [Pixels]	Index	Resolution [Pixels]	Index	Resolution [Pixels]
1	1500 × 687	2	4608 × 3456	3	1500 × 783
4	1500 × 894	5	632 × 1036	6	596 × 747
7	1147 × 767	8	1180 × 998	9	1265 × 1125
10	591 × 1013	11	1288 × 753	12	836 × 868
13	653 × 995				

**Table 12:** Pixel frequency for each class.

Sampling Numbers	Black [%]	Red [%]	Cyan [%]	Yellow [%]	Green [%]
64	2.62	12.29	23.67	45.61	15.82
128	2.72	12.89	22.99	45.48	15.92
256	2.56	13.12	23.13	45.22	15.97
512	2.59	13.08	23.10	45.20	16.04

**Table 13:** Class-wise IoU of each image [%].

Index	Black	Red	Cyan	Yellow	mIoU
13	92.23	15.60	69.74	93.45	67.75
4	94.50	18.53	52.34	93.24	64.65
3	94.85	7.18	56.87	84.00	60.73
7	–	46.42	62.91	74.44	61.26
12	–	76.44	71.71	72.39	55.14
2	71.83	16.46	31.51	86.35	51.54
9	–	36.98	53.22	66.76	52.32
1	91.88	5.28	55.93	46.75	49.96
5	–	48.19	43.13	57.07	37.10
10	79.65	34.85	46.52	41.16	50.54
6	–	53.58	22.28	50.99	31.71
8	–	49.78	66.37	50.90	41.76
11	–	49.35	76.07	20.46	36.74
mean	87.49	35.28	54.51	64.46	50.84

**Table 14:** Correlation between the pixel occupancy ratio and ranking of each image [%].

	Black		Red		Cyan		Yellow		mIoU	
**Index**	**Rate**	**Rank**	**Rate**	**Rank**	**Rate**	**Rank**	**Rate**	**Rank**	**mIoU [%]**	**Rank**
1	6.86	3	32.37	2	0.80	11	32.76	10	48.89	7
2	10.63	1	0.62	12	5.91	13	44.59	7	51.02	6
3	8.86	2	2.31	10	14.87	7	58.54	4	57.97	3
4	3.82	4	0.52	13	7.58	12	74.97	2	62.07	2
5	0.00	–	6.87	8	42.86	3	39.43	8	34.82	12
6	0.00	–	29.98	3	9.59	10	47.78	6	35.03	11
7	0.00	–	8.24	7	21.11	5	58.72	3	54.40	4
8	0.00	–	8.83	6	49.72	2	32.50	11	32.82	13
9	0.00	–	23.52	4	13.89	8	53.67	5	45.71	8
10	0.79	6	2.51	9	64.93	1	27.04	12	35.59	10
11	0.00	–	40.72	1	31.51	4	8.92	13	37.70	9
12	0.00	–	13.44	5	19.42	6	33.62	9	52.37	5
13	2.34	5	0.68	11	11.25	9	75.30	1	65.51	1
mean	2.56	–	13.12	–	23.13	–	45.22	–	–	–

**Table 15:** Compared mIoU for each class [%].

Class	DeepLabv3+	SegFormer
Black	51.57	87.49
red	24.11	35.28
Cyan	44.45	54.51
Yellow	59.17	64.46
mean	42.66	50.84

**Table 16:** Comparison of mIoU for each class [%].

Class	DeepLabv3+	SegFormer
Black	25.66	65.56
Red	8.96	11.30
Cyan	20.12	29.73
Yellow	41.93	54.61
mean	24.17	40.30

**Table 17:** mIoU after re-annotation [%].

Position		DeepLabv3+		SegFormer	
**Row**	**Column**	**mIoU**	Δ	**mIoU**	Δ
1	7	25.96	25.94	16.26	0.87
3	8	18.85	18.64	23.54	20.83
8	2	10.75	10.23	50.09	39.69

## Data Availability

Datasets described as a result of this study are available on request to the corresponding author.

## Abbreviations

The following abbreviations are used in this manuscript:

ASSP	Atrous Spatial Pyramid Pooling
CCS	Carbon Capture and Storage
CNN	Convolutional Neural Network
CO${}_{2}$	Carbon Dioxide
COCO	Common Objects in Context
CUDA	Compute Unified Device Architecture
DM	Diffusion Model
ERF	Effective Receptive Field
FCN	Fully Convolutional Networks
FFN	Feed-Forward Network
GANs	Generative Adversarial Networks
GPU	Graphics Processing Unit
IPCC	Intergovernmental Panel on Climate Change
IoU	Intersection over Union
MLP	Multi-Layer Perceptron
MiT	Mix Transformer
OASIS	Only Adversarial Supervision for Semantic Image Synthesis
PE	Positional Encoding
RoI	Regions of Interest
SETR	SEgmentation TRansformer
SGD	Stochastic Gradient Descent
ViT	Vision Transformer

## Appendix A

Below are the IoU scores for all classes belonging to things and stuff in the COCO-Stuff 10K and ADE20K datasets. These scores indicate the detailed accuracy of both models, DeepLabv3+ and SegFormer, in correctly segmenting the classes. The **bolded numbers** highlight higher scores obtained when comparing both models.

**Table A1:** Class-wise IoU for things in COCO-Stuff10K [%].

Class	DeepLabv3	SegFormer	Class	DeepLabv3	SegFormer
tennis racket	15.76	27.68	surfboard	71.80	70.94
skateboard	26.65	48.34	baseball bat	13.37	30.81
baseball glove	0.00	0.00	kite	45.88	68.52
sports ball	55.70	11.14	snowboard	21.28	45.61
skis	2.78	21.03	frisbee	11.60	44.03
suitcase	25.18	66.25	tie	0.00	2.40
handbag	4.05	8.36	umbrella	62.33	69.19
backpack	5.93	21.17	giraffe	52.74	83.98
zebra	86.91	87.57	bear	37.99	89.50
elephant	59.93	88.81	cow	53.15	90.44
sheep	40.97	84.38	horse	71.81	86.98
cat	55.29	88.63	dog	24.11	77.93
bird	38.15	59.87	bench	29.19	37.20
parking meter	59.48	89.54	traffic light	36.82	70.60
fire hydrant	70.29	87.96	stop sign	73.00	74.41
bicycle	33.22	60.91	car	46.04	44.54
motorcycle	66.16	80.05	airplane	68.95	78.95
bus	70.55	82.24	train	60.39	88.32
truck	35.67	70.61	boat	52.48	63.09
person	78.55	84.16	book	45.09	47.84
clock	35.16	64.99	vase	46.74	48.94
scissors	26.62	54.65	teddy bear	33.03	81.55
hair drier	0.00	0.00	toothbrush	0.00	1.99
microwave	34.35	41.33	oven	50.00	59.42
toaster	0.00	18.69	sink	42.76	58.39
refrigerator	50.54	59.53	tv	42.25	59.01
laptop	36.15	64.43	mouse	45.62	42.46
remote	0.84	66.56	keyboard	17.29	81.95
cell phone	24.06	33.89	chair	33.54	48.63
couch	59.02	59.05	potted plant	35.47	36.99
bed	53.02	63.53	dining table	48.94	58.11
toilet	59.77	83.22	banana	38.59	41.56
apple	1.10	38.13	sandwich	61.63	63.95
orange	25.74	32.78	broccoli	67.00	90.13
carrot	7.92	37.40	hot dog	41.91	42.32
pizza	67.11	81.37	donut	22.61	57.08
cake	17.93	57.46	bottle	42.24	51.86
wine glass	49.48	72.08	cup	32.78	44.00
fork	0.65	19.05	knife	3.51	46.00
spoon	2.49	32.52	bowl	42.07	53.98

**Table A2:** Class-wise IoU for stuff in COCO-Stuff10K [%].

Class	DeepLabv3	SegFormer	Class	DeepLabv3	SegFormer
water-other	19.24	21.55	waterdrops	0.00	0.00
sea	65.57	66.52	river	11.40	44.62
fog	0.00	0.00	ground-other	3.14	9.84
platform	20.64	20.55	playingfield	63.24	63.64
railroad	43.87	53.73	road	51.84	65.22
pavement	42.58	47.46	gravel	2.19	20.74
mud	0.00	2.39	dirt	31.02	40.61
snow	73.16	91.54	sand	50.21	63.95
solid-other	0.00	0.00	hill	27.78	30.05
mountain	28.62	27.21	stone	5.36	0.17
rock	28.32	54.30	wood	2.88	8.85
sky-other	55.20	61.01	clouds	40.09	48.49
plant-other	12.16	25.00	straw	19.70	14.40
moss	0.00	0.00	branch	0.00	0.16
flower	4.71	13.86	bush	17.87	15.57
leaves	4.00	16.76	tree	68.23	75.57
grass	68.18	71.86	structural-other	12.52	12.93
railing	8.90	15.33	net	28.41	37.45
cage	0.00	7.05	fence	36.62	38.94
building-other	51.70	52.49	bridge	8.07	0.62
roof	8.55	2.91	tent	38.95	56.80
skyscraper	9.06	24.91	house	29.26	28.10
food-other	8.13	29.58	fruit	9.43	21.61
vegetable	19.34	32.11	salad	0.00	0.00
textile-other	2.07	11.86	banner	32.09	37.99
blanket	0.00	0.00	pillow	0.00	0.00
cloth	0.00	0.99	clothes	2.17	19.50
curtain	47.46	63.77	towel	16.01	34.49
mat	0.00	6.49	rug	38.87	57.84
napkin	0.00	1.63	furniture-other	8.68	10.82
shelf	5.40	20.65	stairs	15.86	26.10
light	22.23	26.23	counter	18.30	31.64
cupboard	43.17	49.49	cabinet	17.07	13.55
desk-stuff	28.84	36.96	door-stuff	27.93	39.87
table	2.51	18.01	mirror-stuff	25.12	35.67
window-blind	29.20	31.76	window-other	33.33	38.89
floor-marble	0.00	2.84	floor-other	28.67	21.44
floor-stone	0.00	14.96	floor-tile	33.41	43.29
floor-wood	48.12	46.96	carpet	50.39	46.40
ceiling-other	59.20	65.81	ceiling-tile	0.00	1.45
wall-brick	33.96	44.85	wall-concrete	16.80	29.21
wall-other	50.18	58.33	wall-panel	6.01	4.82
wall-stone	15.68	30.08	wall-tile	32.40	51.68
wall-wood	21.83	29.07	cardboard	0.20	12.39
metal	4.25	5.86	paper	5.53	21.37
plastic	0.00	11.73			

**Table A3:** Class-wise IoU for things in ADE20K [%].

Class	DeepLabv3	SegFormer	Class	DeepLabv3	SegFormer
tree	69.39	74.16	bed	84.09	88.08
windowpane	56.51	59.29	cabinet	52.60	60.51
person	71.29	78.94	door	37.36	46.76
table	51.40	60.59	plant	48.27	50.30
curtain	66.07	71.38	chair	48.92	56.77
car	78.68	82.59	painting	59.54	70.40
sofa	59.97	64.29	shelf	34.34	43.31
mirror	57.72	65.78	armchair	35.21	46.35
seat	51.01	64.79	fence	39.88	45.26
desk	42.37	51.99	rock	31.84	36.84
wardrobe	38.05	46.14	lamp	57.51	63.08
bathtub	60.63	74.37	railing	35.41	31.87
cushion	50.82	52.77	base	27.48	27.04
box	15.89	26.15	column	36.92	46.16
signboard	32.91	37.49	chest of drawers	41.70	44.49
counter	20.46	24.83	sink	62.19	67.65
fireplace	63.16	73.39	refrigerator	57.58	78.26
stairs	27.06	28.35	case	42.07	50.71
pool table	79.52	91.59	pillow	51.72	55.75
screen door	53.31	70.11	bookcase	24.73	42.20
blind	34.86	39.10	coffee table	53.47	55.82
toilet	77.77	82.28	flower	24.44	44.49
book	41.04	45.69	bench	40.14	40.26
countertop	52.14	58.24	stove	68.52	76.59
palm	39.55	43.87	kitchen island	27.19	34.10
computer	36.40	65.94	swivel chair	34.71	40.12
boat	25.24	39.25	bar	28.48	44.11
arcade machine	36.14	69.37	bus	56.52	86.24
towel	48.11	62.31	light	43.41	53.20
truck	25.04	34.85	chandelier	60.90	69.86
awning	13.61	25.62	streetlight	21.71	23.86
booth	22.90	52.80	television receiver	64.69	65.75
airplane	24.01	66.39	apparel	24.74	29.85
pole	17.60	23.12	bannister	9.32	11.50
ottoman	44.25	48.14	bottle	10.61	21.03
buffet	27.79	30.45	poster	25.29	25.88
van	26.74	40.40	ship	33.57	63.45
fountain	11.22	20.58	canopy	11.63	23.29
washer	51.52	68.49	plaything	17.54	27.21
stool	26.82	38.10	barrel	11.91	56.82
basket	23.40	33.98	tent	72.27	89.36
bag	4.96	9.14	minibike	63.70	63.94
cradle	72.71	74.44	oven	32.33	58.40
ball	25.58	40.12	food	38.72	15.93
step	2.08	14.52	tank	31.03	53.08
trade name	18.61	29.28	microwave	30.98	86.21
pot	35.62	42.08	animal	48.20	50.62
bicycle	47.71	50.56	dishwasher	59.88	75.42
screen	37.17	61.95	blanket	5.96	12.79
sculpture	31.79	56.36	hood	47.06	70.65
sconce	34.15	43.78	vase	29.11	32.15
traffic light	21.66	32.36	tray	1.21	6.37
ashcan	26.08	41.41	fan	47.26	61.35
crt screen	0.00	11.63	plate	31.14	49.25
monitor	25.07	7.29	bulletin board	22.62	48.28
shower	0.19	2.73	radiator	45.10	58.74
glass	8.70	12.77	clock	20.38	41.62
flag	27.18	62.82			

**Table A4:** Class-wise IoU for stuff in ADE20K [%].

Class	DeepLabv3	SegFormer	Class	DeepLabv3	SegFormer
wall	70.78	76.42	building	77.96	79.87
sky	92.35	93.98	floor	73.83	79.80
ceiling	78.69	83.96	road	79.11	83.36
grass	65.56	69.11	sidewalk	60.75	65.40
earth	31.13	38.42	mountain	51.07	56.70
water	46.40	48.64	house	42.56	31.97
sea	41.07	56.82	rug	48.01	56.01
field	26.18	28.27	sand	25.95	48.70
skyscraper	58.30	49.58	grandstand	32.72	36.39
path	20.01	19.70	runway	58.27	67.16
stairway	32.61	31.64	river	22.90	12.57
bridge	49.25	68.01	hill	8.62	15.33
hovel	17.67	8.90	tower	39.34	6.38
dirt track	3.54	21.65	land	0.02	3.73
escalator	2.68	44.32	stage	7.84	13.89
conveyor belt	42.59	71.14	swimming pool	26.95	55.45
waterfall	46.91	51.06	lake	18.20	58.32
pier	40.95	26.90			
